# Kernel Water Relations and Kernel Filling Traits in Maize (*Zea mays* L.) Are Influenced by Water-Deficit Condition in a Tropical Environment

**DOI:** 10.3389/fpls.2021.717178

**Published:** 2021-10-12

**Authors:** Md. Robiul Alam, Sutkhet Nakasathien, Md. Samim Hossain Molla, Md. Ariful Islam, Md. Maniruzzaman, Md. Akkas Ali, Ed Sarobol, Vichan Vichukit, Mohamed M. Hassan, Eldessoky S. Dessoky, Enas M. Abd El-Ghany, Marian Brestic, Milan Skalicky, S. V. Krishna Jagadish, Akbar Hossain

**Affiliations:** ^1^On-Farm Research Division, Bangladesh Agricultural Research Institute, Pabna, Bangladesh; ^2^Department of Agronomy, Faculty of Agriculture, Kasetsart University, Bangkok, Thailand; ^3^On-Farm Research Division, Bangladesh Agricultural Research Institute, Rangpur, Bangladesh; ^4^On-Farm Research Division, Bangladesh Agricultural Research Institute, Joydebpur, Bangladesh; ^5^Department of Biology, College of Science, Taif University, Taif, Saudi Arabia; ^6^Department of Genetics, Faculty of Agriculture, Menoufia University, Tanta, Egypt; ^7^Department of Plant Physiology, Slovak University of Agriculture, Nitra, Slovakia; ^8^Department of Botany and Plant Physiology, Faculty of Agrobiology, Food and Natural Resources, Czech University of Life Sciences Prague, Prague, Czechia; ^9^Department of Agronomy, Kansas State University, Manhattan, KS, United States; ^10^Bangladesh Wheat and Maize Research Institute, Dinajpur, Bangladesh

**Keywords:** water deficit, maize, kernel water relations, kernel filling traits, stem reserve mobilization

## Abstract

Water deficit is a major limiting condition for adaptation of maize in tropical environments. The aims of the current observations were to evaluate the kernel water relations for determining kernel developmental progress, rate, and duration of kernel filling, stem reserve mobilization in maize. In addition, canopy temperature, cell membrane stability, and anatomical adaptation under prolonged periods of pre- and post-anthesis water deficit in different hybrids was quantified to support observations related to kernel filling dynamics. In this context, two field experiments in two consecutive years were conducted with five levels of water regimes: control (D1), and four water deficit treatments [V10 to V13 (D2); V13 to V17 (D3); V17 to blister stage (D4); blisters to physiological maturity (D5)], on three maize hybrids (Pioneer 30B80, NK 40, and Suwan 4452) in Expt. 1. Expt. 2 had four water regimes: control (D1), three water deficit treatments [V10 to anthesis (D2); anthesis to milk stage (D3); milk to physiological maturity (D4)], and two maize hybrids (NK 40 and Suwan 4452). Water deficit imposed at different stages significantly reduced maximum kernel water content (MKWC), kernel filling duration (KFD), final kernel weight (FKW), and kernel weight ear^–1^ while it increased kernel water loss rate (KWLR), kernel filling rate (KFR), and stem weight depletion (SWD) across maize hybrids in both experiments. The lowest MKWC under water deficit was at D3 in both experiments, indicating that lower KFR results in lowest FKW in maize. Findings indicate that the MKWC (*R*^2^ = 0.85 and 0.41) and KFR (*R*^2^ = 0.62 and 0.37) were positively related to FKW in Expt. 1 and 2, respectively. The KFD was reduced by 5, 7, 7, and 11 days under water deficit at D3, D4 in Expt. 2 and D4, D5 in Expt. 1 as compared to control, respectively. Water deficit at D5 in Expt. 1 and D4 in Expt. 2 increased KWLR, KFR, and SWD. In Expt. 2, lower canopy temperature and electrical conductivity indicated cell membrane stability across water regimes in NK 40. Hybrid NK 40 under water deficit had significantly higher cellular adaptation by increasing the number of xylem vessel while reducing vessel diameter in leaf mid-rib and attached leaf blade. These physiological adjustments improved efficient transport of water from root to the shoot, which in addition to higher kernel water content, MKWC, KFD, KFR, and stem reserve mobilization capacity, rendered NK 40 to be better adapted to water-deficit conditions under tropical environments.

## Introduction

Kernel water relations are a good indicator of kernel developmental progress during grain filling in maize (Schnyder and Baum, 1992; Borras and Westgate, 2006). Developing kernels accumulate more water than assimilate reserves early in the development, and both kernel water content (KWC) and kernel dry matter patterns have been shown to be closely related. As such, understanding kernel water relations is a powerful tool for determining and predicting differences in kernel growth and development among hybrids exposed to different environmental conditions (Saini and Westgate, 2000; Borras and Westgate, 2006; Gambin et al., 2007).

Kernel water relations play a key role in controlling kernel growth and development and the duration of grain filling. The maximum water content achieved early in development provides a fairly accurate estimate of potential kernel size and is closely related to the kernel-growth rate (Borras et al., 2003; Borras and Westgate, 2006). Potential kernel weight can be estimated with maximum kernel water content (MKWC), as final kernel weight (FKW) and kernel water relations are strongly associated in maize (Melchiori and Caviglia, 2008). Kernel water accumulation can be used to mark the progress of kernel development during grain filling (Swank et al., 1987; Schnyder and Baum, 1992; Egli and TeKrony, 1997; Borras and Westgate, 2006). Kernel-filling duration is controlled by the relationship between kernel water and biomass accumulation. Several authors have suggested that water loss from kernels during grain filling is merely an exchange between dry matter and water (Millet and Pinthus, 1984; Brooking, 1990; Saini and Westgate, 2000). Grain filling is the final stage of growth in cereals where ovaries that are fertilized at pollination develop into caryopses. Grain filling duration and rate determine the final grain weight, which is a key component that determines overall yield. Water deficit events during the grain filling stage can cause a major reduction in yield by reducing starch accumulation as a result of limited assimilate partitioning to the developing grain (Blum, 1998) or by direct effects on processes of grain growth (Yang et al., 2004). In the early stages of grain fill, endosperm cells determine the maximum amount of starch and protein that can be accumulated in each kernel (Egli, 1998) as influenced by the rate and duration of grain fill. Water stress during grain filling reduces photosynthesis, induces early senescence, and shortens the grain-filling period, which are more highly affected by water stress than grain-filling rate (Brooks et al., 1982; Schnyder and Baum, 1992; Altenbach et al., 2003; Borras et al., 2003). Westgate and Grant (1989) showed that a brief water deficit during linear grain filling had little impact on dry matter accumulation of maize kernels, but did cause a substantial decrease in KWC.

Water deficit stress decreases kernel filling duration (KFD) (Frederick et al., 1991; De Souza et al., 1997), defined as the time from fertilization to physiological maturity (PM), leading to smaller kernels. Prasad et al. (2008) reported that water deficit occurring after flowering has little effect on kernel-filling rate but shortens KFD, leading to smaller kernel size and less yield. Kernel size is largely dependent on photosynthetic reserves that can be mobilized by the plant. Additional reduction in carbohydrates and nitrogen supply, either from a decrease in photosynthetic activity or a reduction in leaf area, would further decrease kernel size and shorten kernel-filling duration, resulting in smaller kernel (Palta et al., 1994). The shortened kernel-filling duration leading to reduced assimilates under water deficit can be compensated through remobilization of stem reserves during grain filling, which is an important supporting process that can largely compensate grain yield decrease (Bdukli et al., 2007). Drought-induced damages on plant cell membranes lead to imbalanced cellular function. The magnitude of plasma membrane damage due to water deficit can be estimated via ionic secretion measurement (Ferrat and Lovatt, 1999; Khan et al., 2007) which can be a good indicator for cell membrane stability. Aslam et al. (2006) reported that relative cell membrane injury (RCI %) could be used as a reliable selection criterion for water deficit tolerance in maize. Balota et al. (2008) concluded that high canopy temperature depression (CTD) tends to have higher grain yield under dry, hot conditions and, therefore, CTD has been used as a selection criterion to improve adaptation to water deficit and heat.

The information on water relations with kernel growth and development and PM under water deficit during different phenological stages is deemed important for better adaptation and phenotyping of maize hybrids in a drought-prone environment. Simultaneously canopy temperature, cell membrane stability, and anatomical adaptation under short and prolonged periods of water deficit might exhibit great significance on the sustainable adaptation of maize in a water deficit environment. In the above context, two independent experiments were conducted to evaluate the kernel water relations for determining kernel developmental progress, rate, and duration of kernel filling, stem reserve mobilization in maize. In addition, canopy temperature, cell membrane stability, and anatomical adaptation under prolonged periods of pre- and post-anthesis water deficit in different hybrids was quantified to support observations related to kernel filling dynamics.

## Materials and Methods

### Experimental Site

Two field experiments were carried out using a randomized complete block design with split-plot arrangement during two growing seasons of 2010–2011 and 2011–12 at the National Corn and Sorghum Research Center (latitude 14.5° N, longitude 101° E, 360 m above sea level) located in Pak Chong, Nakhon Ratchasima, Thailand. Soils at the experimental site belong to the Pak Chong (PC) soil series. Soil details for the experimental site are available in the authors’ previous publication (Molla et al., 2019).

### Experimental Treatments

**FIGURE 1:**
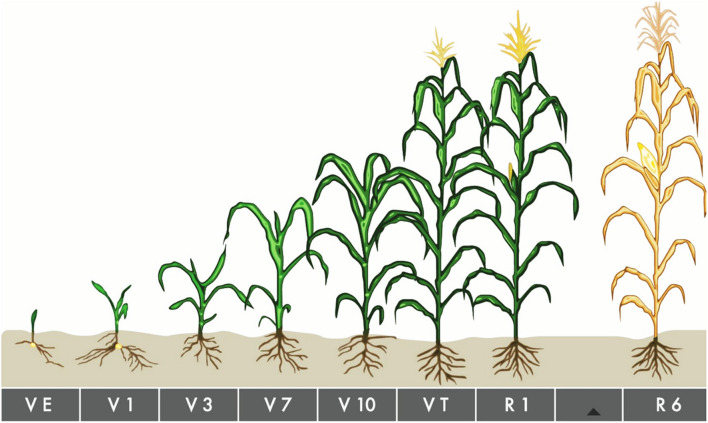
Maize growth stage from emergence to maturity. VE About 4–5 days after planting under ideal conditions, but up to 2 weeks or longer under cool or dry conditions. V1–V5 At V1, round-tipped leaf on first collar appears, nodal roots elongate. By V2, plant is 2–4 inches tall and relies on the energy in the kernel. V3 begins 2–4 weeks after VE and V5, the number of potential number of leaf and ears are determined. V6–V8 Beginning 4–6 weeks after VE. V9–V11 Around 6–8 weeks after VE. V12–Vnth By V12, the plant is about 4 feet tall or more. VT Beginning around 9–10 weeks after emergence. R Corn plants enter reproductive growth after completing tassel emergence, although reproductive stages are determined by kernel development, instead of plant collars. R6 Black Layer Attained about 60 days after silking stage.

**FIGURE 2:**
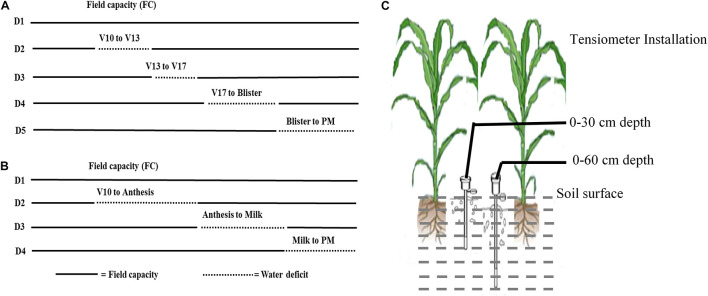
Five water regimes imposed at different growth stages of maize in experiment I **(A)** and four water regimes at different growth stages of maize in experiment II **(B)**. Tensiometers were installed at 0–30 and 0–60 cm depth to monitor soil water potential **(C)**. Sample (-) soil moisture at FC (<–40 KPa) and (—) indicates the period of water deficit.

In Expt. 1, treatments consisted of five water regimes viz., D1 = Control-soil water status maintained near field capacity (FC), D2 = Water deficit from V10 to V13, D3 = Water deficit from V13 to V17 stage, D4 = Water deficit from V17 to blister stage, and D5 = Water deficit from blister to PM as the main plot factor. Three maize hybrids viz., V1 = Pioneer 30B80, V2 = NK 40, and V3 = Suwan 4452 were selected as subplot factor. Characteristics of all maize hybrids used in the study are given in Table 1. Maize growth stage from emergence to maturity are presented in Figure 1 and growth stage details related to the experimental treatments are presented in Figure 2.

**TABLE 1:** Characteristics of maize hybrids used in Expt. 1 and 2. [Source: National Corn and Sorghum Research Center, Thailand (2010)].

Hybrids	Description	Duration (days)	Grain yield (kg ha^–1^)	Released by
Pioneer 30B80	Single cross hybrid Highly adapted to major maize growing areas	100–150	7083	Pioneer Hi-Bred (Thailand) Co., Ltd.
NK 40	Single cross hybrid Widely adapted	105–115	7209	Syngenta Seeds Ltd.
Suwan 4452	Single cross hybrid Widely adapted	100–120	7747	National Corn and Sorghum Research Center

In Expt. 2, four water regimes included D1 = Control–soil water status maintained near FC, D2 = Water deficit from V10 to anthesis, D3 = Water deficit from anthesis to milk stage, and D4 = Water deficit from milk to PM stage as the main plot factor and two higher yielding hybrids from Expt. 1 viz., V1 = NK 40 and V2 = Suwan 4452 were assigned as subplot factor.

The water management for the early establishment of crop was through sprinkler irrigation and continued up to 41 days after planting (before the onset of water-deficit treatments). Water deficit under different treatments was imposed on plants at 42 days after planting (V10 stage) by withholding irrigation for a specific duration (mentioned in treatments) followed by re-watering up to PM stage with weekly flood irrigation. To avoid lateral seepage of water around 7.5 m plot to plot distance and a deep trench was dug between main plots to impose different water-deficit treatments. Two tensiometers were installed at 0–30 cm and 0–60 cm depth to monitor daily soil water tension in the experimental plot throughout the crop cycle. Both experiments were carried out during the winter season which was characterized by very low or no rainfall. Therefore, the experiments were not affected by seasonal rainfall.

### Field and Crop Management

Soil preparation was performed in accordance with conventional approaches at the Research Station, including disk harrow plow followed by leveler. The plot was finally prepared with ridges and furrows maintaining interrow spacing of 75 cm. Mixed fertilizers (N:P = 16:20) were applied at the rate of 156 kg ha^–1^ during final land preparation and properly incorporated with soil. The kernels of each maize hybrid were sown by a manual operated instrument which maintained two kernels hill^–1^ in 10 rows with each row 7.5 m long, with a spacing of 75 and 25 cm between rows and plant, respectively, on December 1, 2010, and January 14, 2011. The following day after sowing, sprinkler irrigation was applied to the plot to ensure uniform germination, and thereafter irrigation was continued at weekly intervals until maize plants reached the knee-high stage. The plants were thinned at the 4-leaf stage to maintain 1 plant hill^–1^. The same amount of mixed fertilizers (as basal) was applied as top-dressing with mechanical applicator at 8–10 leaves stage. Sprinkler irrigation was applied immediately after the top dressing of fertilizers.

### Physiological Parameters

#### Canopy Temperature

In both years, the canopy temperature of leaf was monitored at the milk stage of the crops under control and water-deficit treatments between 11:00 and 12:00 a.m by using a hand-held PCS tester (TOA Electronics Ltd., Japan).

#### Electrical Conductivity of Leaf Tissue

The electrical conductivity (EC) of the leaves was measured at the end of each water-deficit treatment. Twenty leaf disk punches were randomly obtained from young leaves and then put into 20 ml distilled water. Later, these samples were placed in a refrigerator (about 5°C) and after 24 h EC of the leaves was recorded using YOA EC meter (CM 14P, TOA Electronics Ltd., Japan). Eventually, EC of the distilled water (as control) was subtracted from these rates and the EC of leaves under different treatments were obtained.

#### Leaf Anatomy

Ear leaf of maize was collected from control and water-deficit treatments and immediately preserved in a humified box and then transported to the Central Laboratory, Faculty of Agriculture, Kasetsart University (UK) for studying the leaf anatomy. After preparing the slide of leaf mid-rib and attached leaf blade tissue, anatomical view was taken with a digital camera Moticam 2500 (Bettlachstrasse 2, 2540 Grenchen, Switzerland) connected to a microscope Olympus CX21 (Manufacturer: Quality Report Co., Ltd., Thailand).

#### Measurement of Kernel-Related Traits

Measurement of kernel number and kernel weight was done after pollination. Four plants per treatment under control and water deficit were sampled on the day of 50% pollination and at 7-day intervals thereafter. The entire ear with surrounding husks were immediately enclosed in an airtight plastic bag and transported to the lab. Immediately after counting kernel number from the ear of the selected plants, 50 kernels were excised from the ear at floret positions 10–15 from the bottom of the ear within a humidified box. Fresh weight was recorded immediately after sampling, and kernel dry weight (KDW) was determined after drying samples at 70°C for at least 96 h.

Kernel water content was measured throughout kernel development starting from pollination and until kernels reached physiological maturity. Fresh and dry weight was used to calculate KWC (mg kernel^–1^). Kernel water content was calculated as the difference between kernel fresh weight and dry weight. Maximum KWC and maximum dry weight were taken as the maximum value measured for each hybrid by treatment combination during the grain-filling period. The rate of kernel water loss was estimated as the ratio between difference of maximum water content and water content at PM and the duration. The ratio between dry matter and water content in the kernel was calculated at each sampling date as described by Sala et al. (2007).

The average rate of grain filling was determined as maximum grain weight divided by duration (day), assuming grain weight to be zero at anthesis. The duration of grain filling was estimated as the difference between days to PM and days to anthesis.

### Statistical Analysis

The experiment was conducted following a randomized complete block design with a split-plot arrangement and replicated three times. Data were analyzed using MSTATC software (Mstatc, 1990). The significant differences between treatment means were compared with the critical difference at 5% probability level by the Duncan’s Multiple Range Test.

## Results and Discussion

### Kernel Water Content and Kernel Dry Weight

Kernel water relations are a good indicator of kernel growth and development progress during grain filling (Schnyder and Baum, 1992; Borras and Westgate, 2006). Kernel water content and KDW was estimated after anthesis to quantify the progress in kernel development in maize hybrids exposed to water deficit. The developmental dynamics of kernel water and kernel dry mass accumulation under two field experiments (Expt. 1 and Expt. 2) with short and long periods of water deficit at different phenological stages has been highlighted.

**FIGURE 3:**
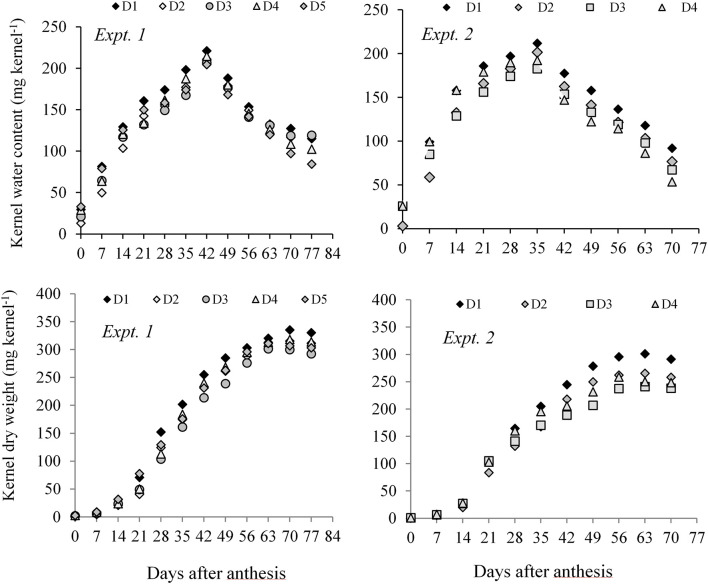
Kernel water content and kernel dry weight of maize hybrids from anthesis to PM at 7 days interval under different water regimes averaged across hybrids in Expt. 1 and Expt. 2. In Expt. 1, D1–Control-soil water status maintained near FC, D2–Water deficit from V10 to V13, D3–Water deficit from V13 to V17 stage, D4–Water deficit from V17 to blister stage, and D5–Water deficit from blister to PM; In Expt. 2, D1–Control–soil water status maintained near FC, D2–Water deficit from V10 to anthesis stage, D3–Water deficit from anthesis to milk stage, and D4–Water deficit from milk to physiological maturity stage.

Both in Expt. 1 and Expt. 2, higher KWC and KDW was sustained in D1 (control) during the grain-filling period. Water deficit at different periods resulted in relatively lower KWC and dry weight measured after anthesis to PM in all three maize hybrids (Figure 3). A general observation was that the KWC under water-deficit condition did not follow a distinct development pattern. However, water deficit at the early vegetative stage gradually sustains a slightly higher water content in the kernel during grain filling as compared to post-anthesis water deficit. Post-anthesis water deficit showed relatively lower kernel water at the later grain-filling stage.

**FIGURE 4:**
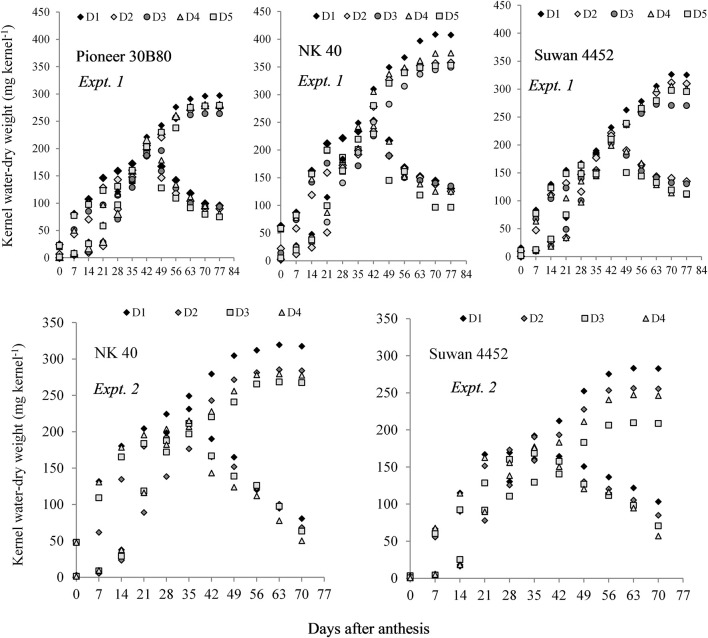
Kernel water content and kernel dry weight of three hybrids (Pioneer 30B80, NK 40, and Suwan 4452) under five water regimes in Expt. 1 and two hybrids (NK 40 and Suwan 4452) under four water regimes in Expt. 2. For treatment details see Figure 3 legend.

**FIGURE 5:**
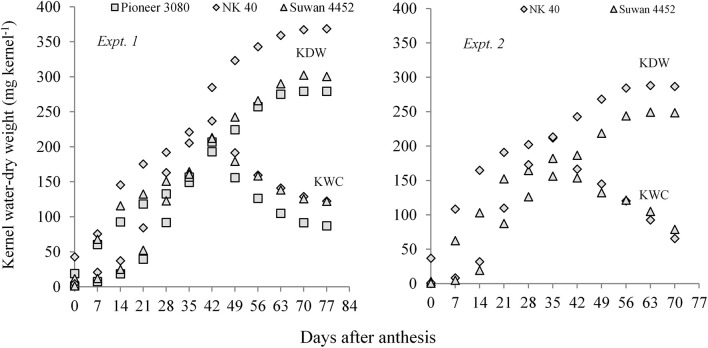
Kernel water content (KWC) and kernel dry weight (KDW) of field maize hybrids after anthesis to physiological maturity at 7 days interval irrespective of water regimes in Expt. 1 and Expt. 2, respectively.

In Expt. 2, water deficit either before anthesis or prolonged water deficit at post-anthesis resulted in lower KDW. This result indicates that water deficit imposed before or after anthesis causes reduced dry mass accumulation in the kernel. All hybrids under different water regimes showed more or less similar response. However, NK 40 contained relatively higher kernel water and KDW under control and water deficit treatments in both experiments as compared to Pioneer 30B80 and Suwan 4452 (Figure 4). Among the hybrids, NK 40 showed its superiority over Suwan 4452 and Pioneer 30B80, based on the progressive development of kernel water and KDW during kernel filling irrespective of water-deficits treatments (Figure 5).

In Expt. 1, water regimes showed significant differences in MKWC. Maximum water content ranged from 225.10 to 206.62 mg kernel^–1^. Well-watered treatment recorded the highest value of MKWC whereas water deficit induced at D3 and D4 accumulated the lowest MKWC (Table 2). Hybrids differed significantly in the maximum water content per kernel achieved during mid grain fill (*P* ≤ 0.05, Table 2).

**TABLE 2:** Effect of water regimes on kernel filling components of maize hybrids in Expt. 1.

Treatments	Maximum water content (mg kernel^–^^1^)	Kernel water loss rate (mg kernel^–^^1^ day^–^^1^)	Kernel filling duration (days)	Kernel filling rate (mg day^–^^1^)
***Water regimes***				
D1	225.10^a^	3.06^c^	72^a^	4.84^b^
D2	214.80^b^	3.98^b^	70^b^	4.94^ab^
D3	206.62^c^	4.11^b^	68^c^	4.38^c^
D4	209.41^c^	4.16^b^	65^d^	4.99^ab^
D5	215.21^b^	5.77^a^	61^e^	5.36^a^
LSD (*P* ≤ 0.05)	5.040	0.482	1.637	0.418
***Hybrids***				
Pioneer 30B80	193.00^c^	4.54^a^	65^b^	4.45^b^
NK 40	236.50^a^	4.27^b^	70^a^	5.52^a^
Suwan 4452	213.21^b^	3.83^c^	66^b^	4.74^b^
LSD (*P* ≤ 0.05)	6.272	0.251	1.511	0.292
***Interaction effect of water regimes and hybrids***				
***D1***				
Pioneer 30B80	202.50^ghi^	3.52^de^	70^cd^	4.15^hi^
NK 40	250.91^a^	3.13^de^	75^ab^	5.78^ab^
Suwan 4452	221.90^cdef^	2.54^fg^	72^*bc*^	4.60^efghi^
***D2***				
Pioneer 30B80	198.11^hij^	4.87^c^	63^e^	5.11^bcdef^
NK 40	233.60^bc^	4.83^c^	76^a^	5.45^abc^
Suwan 4452	212.70^efgh^	2.23^g^	71^cd^	4.26^ghi^
***D3***				
Pioneer 30B80	186.40^f^	3.56^de^	62^ef^	3.89^i^
NK 40	225.40^cde^	3.04^ef^	68^d^	4.93^cdefg^
Suwan 4452	208.12^fgh^	5.72^b^	73^abc^	4.31^ghi^
***D4***				
Pioneer 30B80	190.50^ij^	5.33^bc^	68^d^	4.40^fghi^
NK 40	228.82^cd^	3.74^d^	70^cd^	5.43^abc^
Suwan 4452	208.91^fgh^	3.40^de^	57^g^	5.15^bcde^
***D5***				
Pioneer 30B80	187.30^ij^	5.43^bc^	63^e^	4.69^defgh^
NK 40	243.73^ab^	6.62^a^	62^ef^	6.03^a^
Suwan 4452	214.62^defg^	5.27^bc^	59^fg^	5.36^abcd^
LSD (*P* ≤ 0.05)	14.02	0.562	3.378	0.901
CV (%)	3.84	7.81	2.95	7.82

*FC, field capacity; PM, physiological maturity.*

*Treatment abbreviated as in Figure 3.*

*Means in each column with the same letter are not significantly different from each other at *p* ≤ 0.05.*

**FIGURE 6:**
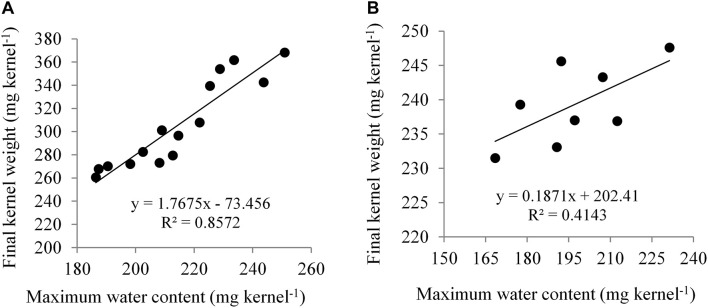
Relationship between maximum kernel water content (MKWC) and final kernel weight (FKW) in Expt. 1 **(A)** and Expt. 2 **(B)**.

NK 40 achieved significantly higher MKWC during the mid grain-filling phase, compared to the average of the other two hybrids. The hybrid Pioneer 30B80 attained the lowest amount of MKWC. It indicates that water shortage before the anthesis stage causes more reduction in water accumulation in Pioneer 30B80 kernels. Interaction of hybrid and water regimes revealed that NK 40 accumulated significantly higher maximum water content in control (D1) compared to water deficit stress treatments, except D5. NK 40 consistently accumulated higher maximum water content as compared to Pioneer 30B80 and Suwan 4452, across all water regimes. Maximum KWC exhibited a significant positive relationship with FKW (Figure 6A). Borras et al. (2003) and Gambin et al. (2007) also reported a significant positive relationship between MKWC and FKW.

In Expt. 2, the maximum water content of the kernel also varied significantly due to water regimes. Maximum water content per kernel ranged from 211.81 to 182.80 mg kernel^–1^ (Table 3). Control and water deficit imposed at milk to physiological stage had the highest value of maximum water content whereas water deficit from anthesis to milk stage recorded the lowest MKWC (Table 3). After anthesis, water deficit up to milk stage limits water uptake by the crops and the subsequent effect would have resulted in lower accumulation of water by the kernel as compared to control or water deficit at other stages. Hybrids showed significant variation in maximum water content (MWC) per kernel (*P* ≤ 0.05, Table 3). NK 40 exhibited significantly higher MKWC as compared to Suwan 4452. NK 40 accumulated significantly higher MKWC across the water regimes as compared to Suwan 4452 (Table 3) suggesting this hybrid has the potential to accumulate higher KWC in control and water-deficit environments. Similar to Expt. 1, MKWC exhibited a positive relationship with FKW (Figure 6B).

**TABLE 3:** Effect of water regimes on grain filling components of maize hybrids in Expt. 2.

Treatment	Maximum water content (mg kernel^–1^)	Kernel water loss rate (mg kernel^–1^ day^–1^)	Kernel filling duration (days)	Kernel filling rate (mg day^–1^)
***Water regimes***				
D1	211.81^a^	2.72^d^	69.50^a^	4.19
D2	192.32^bc^	3.44^c^	64.35^ab^	4.34
D3	182.80^c^	3.88^b^	64.00^b^	3.72
D4	201.60^ab^	4.07^a^	62.50^b^	4.06
LSD (*P* ≤ 0.05)	11.34	0.178	5.183	ns
***Hybrids***				
NK 40	212.03^a^	4.40^a^	66	4.44^a^
Suwan 4452	182.21^b^	2.65^b^	65	3.71^b^
LSD (*P* ≤ 0.05)	23.75	0.97	ns	0.492
***Interaction effect of water regimes and hybrids***				
***D1***				
NK 40	231.30^a^	3.54^bc^	72^a^	4.27^ab^
Suwan 4452	192.20^bc^	1.90^d^	67^b^	4.10^ab^
***D2***				
NK 40	207.20^ab^	4.21^ab^	65^bc^	4.90^a^
Suwan 4452	177.50^bc^	2.67^cd^	64^bc^	3.77^ab^
***D3***				
NK 40	197.10^abc^	4.80^a^	64^bc^	4.26^ab^
Suwan 4452	168.40^c^	2.95^bcd^	64^bc^	3.18^b^
***D4***				
NK 40	212.40^ab^	5.05^a^	62^c^	4.32^ab^
Suwan 4452	190.70^bc^	3.08^bcd^	63^bc^	3.86^ab^
LSD (*P* ≤ 0.05)	33.40	1.191	3.706	1.284
CV (%)	9.00	17.94	3.02	16.73

*FC, field capacity; PM, physiological maturity.*

*Treatment abbreviated as in Figure 3.*

*Means in each column with the same letter are not significantly different from each other at *P* ≤ 0.05.*

### Loss of Kernel Water

For maize kernel growth, the effective grain-filling period is more important for active dry matter accumulation and actual kernel size determination (Borras et al., 2009). During this phase, KWC reaches its maximum value and then begins to decline closely coordinated with dry matter deposition. A difference in the duration of kernel filling is a consequence of variations in the relationship between kernel water loss and dry matter accumulation after attaining maximum water content. In the present study, the water loss rate from the kernel was determined after attaining maximum water content.

In Expt. 1, water regimes showed significant differences in dynamics of kernel water loss after attaining maximum water content. The maximum rate of water loss per day from the kernel (5.77 mg kernel^–1^ day^–1^) was observed in D5 treatment. The control showed a significantly lower rate of water loss per day compared to the average of all the water-deficit treatments (Table 2). This result indicates that water deficit imposed during grain filling increases the rate of water loss as compared to water deficit at vegetative stage and control. Hybrids also exerted significant variation on kernel water loss per day. Pioneer 30B80 showed the maximum rate of kernel water loss (4.54 mg kernel^–1^ day^–1^) while Suwan 4452 demonstrated the minimum rate (3.83 mg kernel^–1^ day^–1^) (Table 2).

The rate of kernel water loss varied across water regimes and the maize hybrids. The kernel water loss rate of NK 40 at D5 was significantly higher, and the lowest was recorded with Suwan 4452 at D2 stage followed by Suwan 4452 at control (Table 2). It was observed that the genotypic response across water regimes did not follow a distinct pattern with the rate of water loss from the kernel after attaining maximum water content. Variation of assimilate availability generally regulates the kernel water uptake and expansion in maize hybrids (Borras et al., 2003; Westgate et al., 2004). It has been investigated that source reduction during the effective grain-filling period accelerates the rate of water loss from kernels, accelerates desiccation, and reduces the grain-filling duration without affecting the biomass accumulation rate (Barlow et al., 1980; Brooks et al., 1982; Jones and Simmons, 1983; Egli, 1990; Westgate, 1994).

In Expt. 2, the maximum rate of water loss per day from the kernel (4.07 mg kernel^–1^ day^–1^) was observed in water deficit at D4 stage. The control recorded significantly lowest rate of water loss per day (2.72 mg kernel^–1^ day^–1^) (Table 3). This result indicates that water deficit imposed after anthesis and successive stages enhance the rate of water loss, reaches peak rate of water loss leading to water shortage after milk stage as compared to water deficit at vegetative stage and control. Hybrids also exerted significant variation on kernel water loss per day. NK 40 showed a significantly higher kernel water loss rate per day compared to Suwan 4452 (Table 3).

**FIGURE 7:**
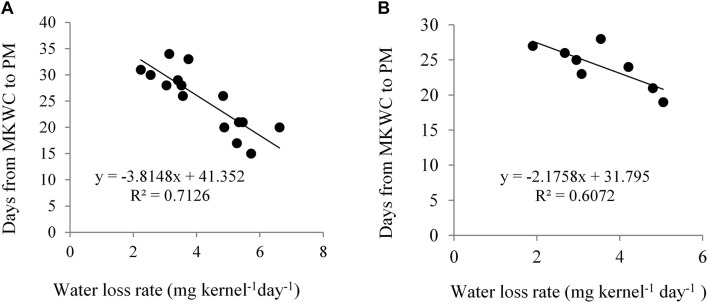
Relationship between kernel water loss rate and days from maximum kernel water content (MKWC) to physiological maturity (PM) in Expt. 1 **(A)** and Expt. 2 **(B)**.

Kernel water loss rate significantly differed across water regimes and the hybrids. NK 40 demonstrated a significantly higher rate of kernel water loss per day under different periods of water deficit as compared to Suwan 4452 (Table 3). Kernel water loss rate exhibited a significant negative relationship with the days from maximum KWC to PM stage in both experiments (Figure 7). Similar findings have also been reported by Westgate (1994) and Pepler et al. (2006).

### Rate and Duration of Kernel Filling

In Expt. 1, water deficit imposed at different stages of crop showed significant influence on grain filling duration and the rate of grain filling. Water management at FC (control) demonstrated the maximum duration of grain filling (72 days) whereas the minimum grain filling duration (61 days) was with water-deficit stress imposed at D5 stage (Table 2). It was generally observed that water deficit at grain-filling stages had a more negative impact than that of vegetative stages, in terms of grain-filling duration. Grain filling was shortened by 2, 4, 7, and 11 days under water deficit from D2, D3, D4, and D5 stages, respectively, as compared to control. Water deficit at reproductive stages shortens the grain-filling duration because of inhibition of current photosynthetic assimilates, i.e., limited source capacity. The rate of grain filling was also significantly affected by water deficit treatments. The highest rate of grain filling was recorded in D5 and D4 stages. Control and D2 stages exhibited the lowest rate of grain filling. Hybrids showed a significant variation in duration and rate of grain filling in maize. NK 40 exhibited a significantly higher duration of grain filling as compared to Pioneer 30B80 and Suwan 4452. A similar result was obtained in the case of rate of grain filling (Table 2). The higher grain filling duration accompanied by a higher rate of grain filling made this hybrid attain higher yield potential. Irrespective of water deficit at different stages of the crop, NK 40 demonstrated superiority over two other maize hybrids. Potential physiological traits such as higher relative water content, chlorophyll content, MKWC, dry matter translocation, and translocation efficiency of NK 40 could have resulted in optimum grain filling and higher grain yield (Molla et al., 2019).

The results indicated that maximum grain-filling duration was attained by NK 40 at D1, D2, and D4 stages. Generally, Pioneer 30B80 recorded relatively lower grain-filling duration under control and stress periods as compared to NK 40 and Suwan 4452, except in D4 and D5 compared to Suwan 4452 (Table 2). Under optimum irrigation condition and water deficit at the early grain-filling stage, NK 40 exhibited its potential capacity to fill the kernel for a relatively long period as compared to other hybrids. The rate of grain filling recorded a significant difference due to water deficit and hybrids interaction. The grain filling rate of NK 40 at D5 was significantly higher which was statistical similar to NK 40 at D1, D2, and D4, and the lowest grain filling rate of Pioneer 30B80 was recorded at D3. In general, NK 40 showed higher efficiency of grain filling under optimum water management and water-deficit conditions imposed at different stages.

**FIGURE 8:**
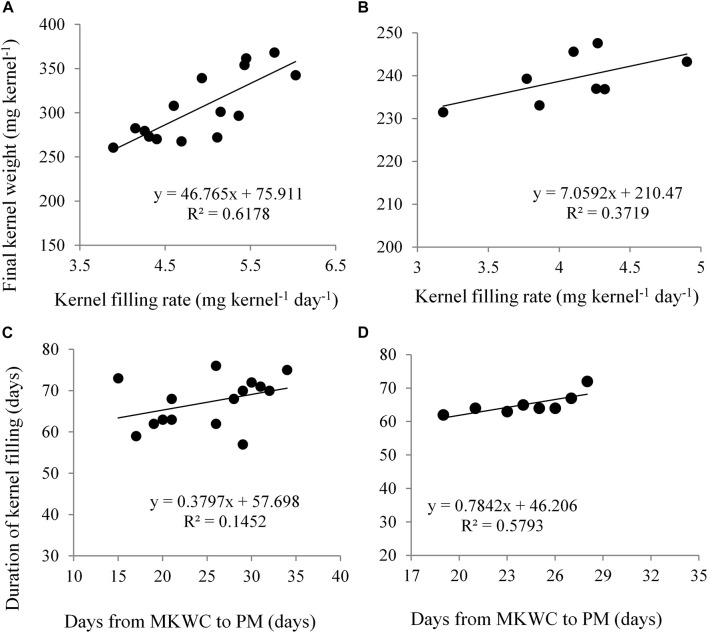
Relationship between kernel filling rate and final kernel weight in Expt. 1 **(A)** and Expt. 2 **(B)**; days from maximum kernel water content (MKWC) to physiological maturity (PM) and duration of kernel filling in Expt. 1 **(C)** and Expt. 2 **(D)**.

In Expt. 2, the highest duration of grain filling (69.50 and 64.53 days) was noted in control and water deficit from V10 to anthesis stage, respectively (Table 3). Water deficit from D3 and D4 stage recorded the lowest duration of grain filling in maize. This result indicated that water deficit at the vegetative stage has a similar response in grain filling as in control. Control condition and water deficit during vegetative stages do not restrict source capacity for grain filling compared to during grain filling. Water deficit during grain-filling stages reduces assimilate supply to the growing kernel which results in shortened duration of grain filling. Severe moisture deficit in soil did not have a significant influence on the rate of grain filling (Table 3). The duration of grain filling was not influenced by the hybrids. The rate of grain filling was found significantly higher in NK 40 as compared to Suwan 4452 (Table 3). Under both water deficit conditions, the higher grain filling rate of NK 40 indicates that it has the potentiality to supply assimilate for meeting the demand of growing kernel. Kernel filling rate (KFR) exhibited a positive relationship with FKW in both experiments (Figures 8A,B). Days from MKWC to PM showed a positive relationship with the duration of kernel filling (Figures 8C,D).

### Kernel Water Content-Dry Weight Ratio

**FIGURE 9:**
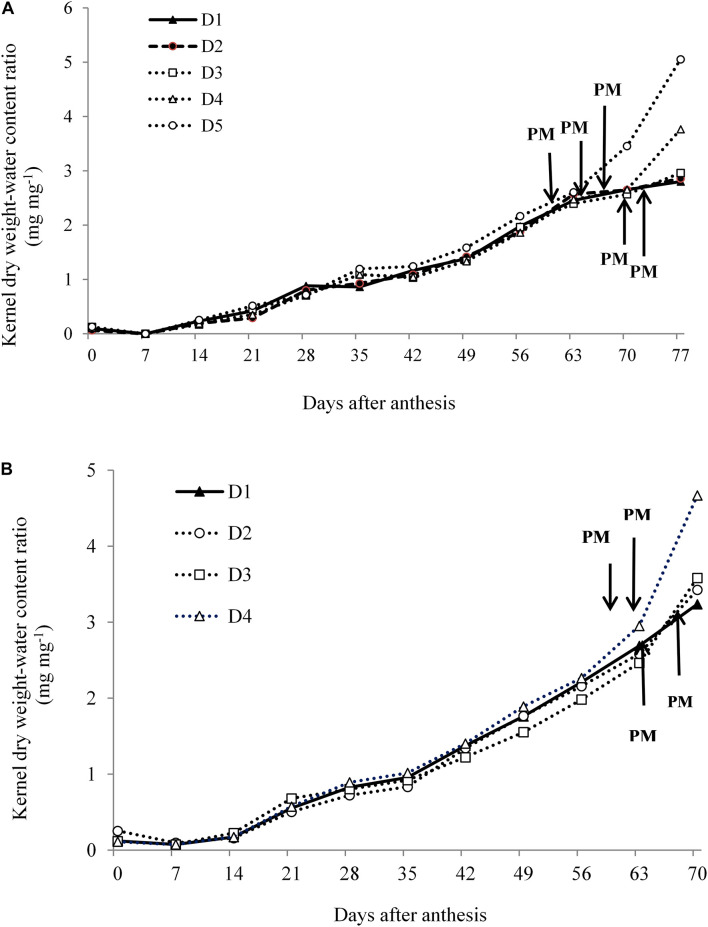
Kernel dry weight-water content ratio under different water regimes at 7 days intervals from days after anthesis to physiological maturity in Expt. 1 **(A)** and Expt. 2 **(B)**.

In Expt. 1, dry weight-KWC ratio under water deficit is a coordinated process and is considered important for evaluating the dynamics of dry matter deposition during effective grain filling. Figure 9A indicates that KDW-water content ratio among water deficit treatments including control was similar during the kernel filling period until they reached the PM stage. After reaching PM, i.e., D5 and D4 treatments, the dry weight-KWC started to increase rapidly than that of D1. This surprising increase in the dry weight-KWC was associated with a decline in KWC after cessation of kernel growth (Figure 8A). Water deficit during reproductive stages (D5, D4, and D3 treatments) limits assimilate supply which eventually shortens grain filling duration by advancing PM, and this result is consistent with rapid increases of dry weight-KWC ratio. Sala et al. (2007) also reported that the dry weight-water content ratio of kernel during grain filling of maize germplasm was affected by the source-sink relationship. In Expt. 2, KDW-water content ratio was quite similar for all treatments, and after attaining PM this ratio sharply increased in case of D4 treatment. This result suggests that limited assimilate supply shortens kernel filling by advancing PM and increasing dry weight-KWC ratio that leads to cessation of kernel growth (Figure 9B).

### Kernel Weight and Stem Reserve Mobilization

In Expt. 1, the FKW was significantly different under water regimes and hybrids. All hybrids attained higher kernel weight under control condition. Kernel weight under control was significantly higher in NK 40, compared to the average of other two hybrids, indicating higher yield potential with increased kernel weight in NK 40 (Table 4). Reduction in FKW was higher in moderate water deficit at D3 stage for all hybrids with a maximum of 11.27% in Suwan 4452 followed by the same hybrid at D2 stage (Table 4). Water deficit at D5 stage recorded slightly higher reduction in kernel weight compared to D4 stage in all hybrids. Kernel weight per ear in the control was significantly higher in Suwan 4452 than NK 40 and Pioneer 30B80, indicating a relatively higher potential ear productivity in Suwan 4452. Reduction in kernel weight per ear was higher in all hybrids at the water deficit imposed from D3 with a higher reduction (21.03%) in Suwan 4452 followed by D2 stage in the same hybrid (19.66%).

**TABLE 4:** The effect of water regimes and hybrids on final kernel weight, kernel weight reduction, kernel weight ear^–1^, reduction in kernel weight ear^–1^, and stem weight depletion (% of kernel weight ear^–1^) in Expt. 1.

Treatment	Final kernel weight (mg kernel^–1^)	Reduction in kernel weight (%)	Kernel weight ear^–1^ (g)	Reduction in kernel weight ear^–1^ (%)	Stem weight depletion (% of kernel weight ear^–1^)
***Water regimes***					
D1	319.50^a^	–	180.60^a^	–	1.730^e^
D2	304.40^c^	4.73	158.40^cd^	6.95	2.343^d^
D3	291.10^d^	8.89	152.60^d^	8.76	5.090^c^
D4	308.50^b^	3.44	168.80^b^	3.69	6.130^a^
D5	302.30^c^	5.38	162.60^bc^	5.63	5.310^b^
LSD (*P* ≤ 0.05)	3.380		6.832		0.203
***Hybrids***					
Pioneer 30B80	270.70^c^	–	162.20^b^	–	3.474^b^
NK 40	353.20^a^	–	167.80^a^	–	5.510^a^
Suwan 4452	291.70^b^	–	163.80^b^	–	3.378^b^
LSD (*P* ≤ 0.05)	5.794		3.685		0.138
***Interaction effect of water regimes and hybrids***					
***D1***					
Pioneer 30B80	282.50^f^	–	176.85b	–	1.36^i^
NK 40	368.23^a^	–	177.59b	–	1.86^h^
Suwan 4452	307.94^e^	–	187.51a	–	1.97^h^
***D2***					
Pioneer 30B80	272.11^fgh^	3.68	158.63^ef^	10.30	2.02^h^
NK 40	361.70^ab^	1.77	166.06^cde^	6.49	3.56^g^
Suwan 4452	279.53^fg^	9.22	150.65^fg^	19.66	1.45^i^
***D3***					
Pioneer 30B80	260.72^h^	7.72	149.38^g^	15.53	4.04^f^
NK 40	339.41^d^	7.82	160.20^de^	9.79	5.28^d^
Suwan 4452	273.23^fgh^	11.27	148.07^g^	21.03	5.95^c^
***D4***					
Pioneer 30B80	270.34^fgh^	4.32	166.78^cde^	5.69	5.31^d^
NK 40	354.13^bc^	3.83	170.68^bc^	3.89	9.01^a^
Suwan 4452	301.21^e^	2.18	168.97^bcd^	9.89	4.08^f^
***D5***					
Pioneer 30B80	267.80^gh^	5.20	159.34^e^	9.90	4.64^e^
NK 40	342.52^cd^	6.98	164.62^cde^	7.30	7.85^b^
Suwan 4452	296.71^e^	3.64	163.71^cde^	12.69	3.44^g^
LSD (*P* ≤ 0.05)	12.960		8.239		0.309
CV (%)	2.49		2.94		4.40

*FC, field capacity; PM, physiological maturity.*

*Treatment abbreviated as in Figure 3.*

*Means in each column with the same letter are not significantly different from each other at *p* ≤ 0.05.*

In general, a decrease in kernel weight per ear under water deficit at different stages was found to be relatively low in NK 40. This could be due to the supplementation of assimilates from the stem reserves when current photosynthesis is limited during water deficit. Moderate water deficit during D3 stage, i.e., before flowering results in a higher reduction in the kernel weight. Reduction in kernel setting might be one of factor that can be attributed to this response. Stem weight depletion (SWD) at D4 and D5 stages in NK 40 was significantly higher as compared to other hybrids. Pioneer 30B80 exhibited a similar pattern of SWD at different stages as in NK 40 but with significantly lower values. Higher depletion of stem weight in Suwan 4452 was noted with water deficit from D3 stage. Stem dry weight depletion as a percentage of grain weight per ear in all hybrids tended to increase under-water deficit imposed at different stages as compared to control (Table 4). However, the SWD and the potential increase in assimilate translocation to grains was higher in NK 40 than other two hybrids.

In Expt. 2, FKW and kernel weight per ear in controls (Table 5) were lower as compared to Expt. 1. This could be associated to climatic variation (delayed planting of 2nd experiment). The first experiment was planted in the first week of December while the prevailing temperature was quite low, whereas the 2nd experiment was planted in mid-January with comparatively higher temperature. So the growth and development of the crop in the 2nd experiment was relatively faster than in Expt. 1. However, NK 40 still maintained its superiority over Suwan 4452 in potential kernel weight whereas Suwan 4452 demonstrated higher productivity in kernel weight per ear over NK 40 only in the control condition. Water deficit at the grain-filling stage exhibited higher reduction in kernel weight in both hybrids (Table 5). Under severe water deficit the reduction in kernel weight was comparatively higher in Suwan 4452 than in NK 40 (Table 5).

**TABLE 5:** The effect of water regimes and hybrids on final kernel weight, kernel weight reduction, kernel weight ear^–1^, reduction in kernel weight ear^–1^, and stem weight depletion (% of kernel weight ear^–1^) in Expt. 2.

Treatment	Final kernel weight (mg kernel^–1^)	Reduction in kernel weight (%)	Kernel weight ear^–1^ (g)	Reduction in kernel weight ear^–1^ (%)	Stem weight depletion (% of kernel weight ear^–1^)
***Water regimes***					
D1	246.60^a^	–	162.40^a^	–	16.80^c^
D2	241.30^a^	2.15	136.80^b^	15.76	7.26^d^
D3	234.30^b^	4.99	120.90^c^	25.55	33.86^b^
D4	235.00^b^	4.70	131.10^b^	19.27	46.74^a^
LSD (*P* ≤ 0.05)	5.903		9.459		1.240
***Hybrids***					
NK 40	241.27	–	141.91	–	33.80
Suwan 4452	237.42	–	133.68	–	18.54
LSD (*P* ≤ 0.05)					
***Interaction effect of water regimes and hybrids***					
***D1***					
NK 40	247.60^a^	–	159.33^a^	–	26.15^c^
Suwan 4452	245.70^ab^	–	165.53^a^	–	7.45^e^
***D2***					
NK 40	243.30^ab^	1.74	147.36^b^	7.51	7.91^e^
Suwan 4452	239.30^ab^	2.60	126.15^d^	23.79	6.61^e^
***D3***					
NK 40	237.00^ab^	4.28	124.38^d^	21.94	49.85^a^
Suwan 4452	231.50^b^	5.78	117.45^d^	29.05	17.88^d^
***D4***					
NK 40	236.90^ab^	4.32	136.53^c^	14.31	51.27^a^
Suwan 4452	233.10^ab^	5.13	125.59^d^	24.13	42.20^b^
LSD (*P* ≤ 0.05)	13.72		9.461		3.354
CV (%)	3.05		3.65		6.81

*FC, field capacity; PM, physiological maturity.*

*Means in each column with the same letter are not significantly different from each other at *p* ≤ 0.05.*

Imposing water stress at anthesis to milk stage accounted for 29.05 and 21.94% kernel weight reduction per ear in Suwan 4452 and NK 40, respectively. In Expt. 2, severe water deficit at D3 accounts for higher kernel weight reduction probably due to higher kernel abortion. Higher SWD was recorded at D4 stage in both hybrids. Both hybrids showed similar pattern of SWD with a higher overall reduction in NK 40.

Stem weight loss under water deficit at D3 and D4 stages accounted for 49.85 and 51.27% of grain weight per ear in NK 40 followed by 42.20% at D4 in Suwan 4452. This finding indicates that stem dry matter reserves may have accounted for about half of the grain weight per ear in NK 40 under severe water-deficit condition, while it was lower in Suwan 4452 (Table 5). Clearly, the contribution of stem reserves to grain mass was consistently higher in NK 40 than in other hybrids under both experiments, indicating an efficient and stable assimilate translocation despite inter annual differences in climatic conditions.

### Canopy Temperature and Cell Membrane Stability Under Water Deficit

**FIGURE 10:**
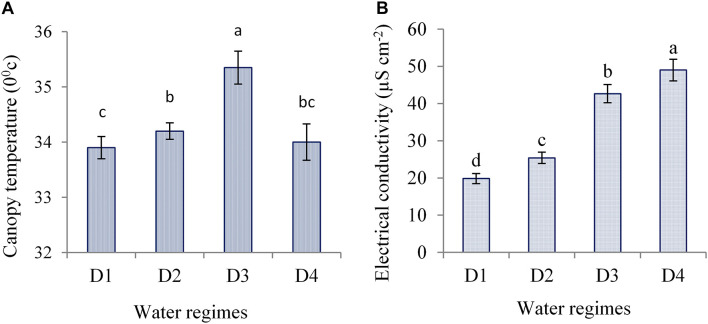
Canopy temperature **(A)** and electrical conductivity **(B)** of ear leaf of maize measured during mid grain filling as affected by water regimes in Expt. 2. Different letters on bars are significantly different from each other at *p* ≤ 0.05.

Canopy temperature varied significantly due to water regimes. Water deficit imposed at anthesis to milk showed significantly higher canopy temperature (CT) as compared to other water-deficit periods and control (Figure 10A). However, control under well-watered condition exhibited the lowest CT. This suggests that optimum water management helps regulate water uptake by roots and induce efficient transpiration cooling through the stomatal regulation leading to lower CT. Conversely, during the periods of water deficit, water shortage in the root zone induces stomatal closure and inhibits water loss through transpiration leading to relatively higher CT. Canopy temperature also differed significantly due to the interaction of hybrids and water regimes. NK 40 and Suwan 4452 showed significantly lower CT under control (33.40 and 34.40°C) and higher (34.80 and 35.90°C) in D3 treatment of Expt. 2. NK 40 recorded significantly lower CT under control and all water deficit treatments as compared to Suwan 4452, indicated that NK 40 sustained relatively higher RWC (%) under control and water-deficit conditions and we hypothesize this to be associated with partially opening of stomata resulting in continued transpirational cooling.

Water-deficit stress causes damages to plasma membranes of plant cell and results in leaking of electrolytes from the cell. The magnitude of plasma membrane damage due to water deficit can be estimated via measuring the proportion of ion leakage (Khan et al., 2007). In the present study, the electrolyte leakage in terms of EC was monitored at the late grain-filling stage of maize exposed to severe water deficit. Water deficit at different stages significantly increased EC as compared to control (Figure 10B). Hybrids showed a significant variation on EC across water regimes. Both hybrids showed significantly lower EC under control as compared to water deficit treatments. NK 40 exhibited relatively lower EC under severe water deficit, which indicated lower membrane damage as compared to Suwan 4452. It has been suggested that the critical feature of tolerance to dehydration depends on the ability of the plant to limit membrane damage during dehydration and to regain membrane integrity and membrane-bound activities quickly upon rehydration (Tripathy et al., 2000). This result suggests that NK 40 showed relatively more water-deficit tolerance than Suwan 4452. Blum and Ebercon (1981) also reported that the changes in membrane permeability following exposure to water-deficit stress can be used to estimate water-deficit tolerance in crop plants.

### Changes in Leaf Anatomy

**FIGURE 11:**
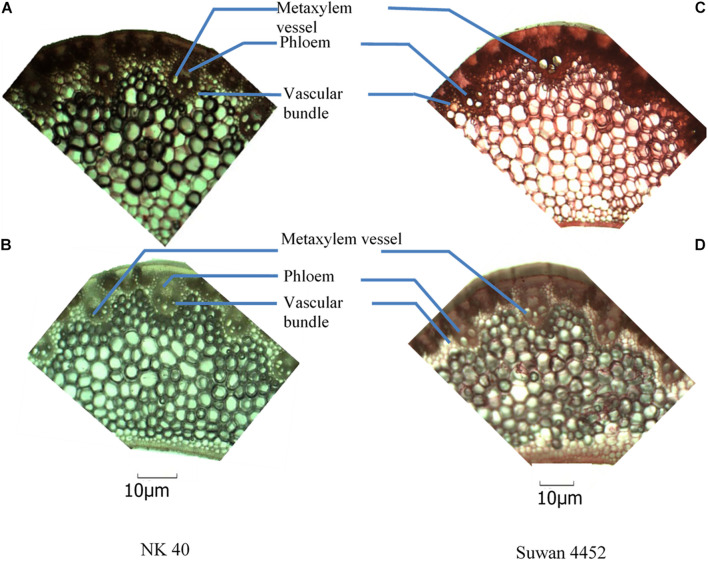
Anatomical view of leaf blade attached to the mid-rib of NK 40 and Suwan 4452 under control **(A,C)** and water deficit **(B,D)** during mid grain-filling stage.

**FIGURE 12:**
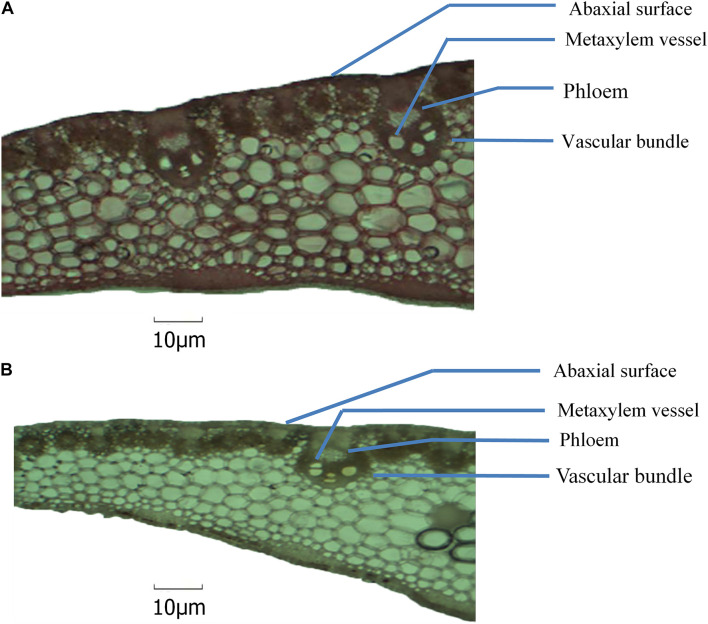
Anatomical view of leaf blade attached with mid-rib of NK 40 under control **(A)** and water deficit **(B)** after milking stage.

In Expt. 2, severe water deficit induced some changes in mid-rib of leaf and attached leaf blade of NK 40 and Suwan 4452. From the anatomic view, it clearly shows that the number of xylem vessel in leaf blade increased under water deficit relative to control treatment in both maize cultivars (Figures 11, 12). The increase in the number of xylem vessels under water deficit is quite consistent in NK 40 compared to Suwan 4452. In contrast, a decrease in the diameter of xylem vessels was observed under water deficit, with higher xylem diameter observed under control. De Souza et al. (2010) also reported a decrease in diameter of metaxylem of maize leaf under water stress condition. The decrease in the diameter of the metaxylem vessel is an adaptation mechanism by the plant under water deficit to avoid cavitation. This change in leaf anatomy is also supported by other studies which have reported that vessels with greater caliber are more prone to cavitation than vessels with smaller caliber (Vasellati et al., 2001). Mostajeran and Rahimi-Eechi (2008) reported that the cavitation process can diminish the plant capacity in transporting water and fixing carbon. A decrease in metaxylem vessel diameter can avoid cavitation and embolism due to the increase in the number of water molecules in contact to xylem cell walls per unit water volume (Hacke and Sperry, 2001). So the increase in the number of metaxylem vessels with narrow diameter could protect the water transportation system, possibly by favoring the water absorption in the root, the flow, and the distribution of water in the leaves.

The relative area of phloem in leaf mid-rib was found to be slightly higher in NK 40 under water deficit compared with control (Figures 11A,B). Though the phloem area in Suwan 4452 is not well visualized in anatomic view, under water deficit phloem area is quite evident. Comparing Suwan 4452, NK 40 exhibited relatively higher phloem area under water deficit indicating more mobilization of photosynthates during grain filling, supporting the higher SWD in both experiments (Tables 4, 5). Souza et al. (2010) also revealed an increased phloem thickness in maize leaf under water stress. A relatively higher number of vascular bundle with smaller inter vascular bundle distance in mid-rib of both hybrids was observed under water deficit as compared to control. It was also an evidence of adaptation under water deficit to maintain a continuous stream of water.

In summary, the major physiological differences that rendered NK 40 to be more tolerant during post-anthesis water-deficit stress compared to other tested hybrids could be due to NK 40 having lower canopy temperature and EC indicated cell membrane stability across, higher kernel water, KDW, and stem reserve mobilization capacity as compared to Pioneer 30B80 and Suwan 4452 across water regimes in both experiments. Under water deficit at milk to PM, NK 40 had significantly higher cellular adaptation by increasing the number of xylem vessel while reducing vessel diameter in leaf mid-rib and attached leaf blade. These physiological adjustments improved efficient transport of water from root to the shoot, which in addition to higher kernel water content, MKWC, KFD, KFR, and stem reserve mobilization capacity, rendered NK 40 to be better adapted to water-deficit conditions under tropical environments.

## Conclusion

Water deficit at different phenological stages exhibited significant variation on kernel water and kernel filling traits of field-grown maize. Water deficit at different periods resulted in relatively lower KWC and KDW measured after anthesis to PM, irrespective of hybrids. Water deficit advanced PM by reducing KFD by 5, 7, 7, and 11 days when maize plants were subjected to water deficit at D3 and D4 in Expt. 2, D4 and D5 in Expt.1, respectively, as compared to control. NK 40 had higher kernel water, KDW, and stem reserve mobilization capacity as compared to Pioneer 30B80 and Suwan 4452 across water regimes in both experiments. In addition, water deficit at milk to PM increased the number of xylem vessel in leaf mid-rib attached leaf blade of NK 40 relative to control which favored better adaptation under water-deficit environment. In addition, imporved vascular physiology, higher kernel water content, MKWC, KFD, KFR, and stem reserve mobilization capacity, helped NK 40 to be better adapted to water-deficit conditions under tropical environments.

## Data Availability Statement

The original contributions presented in the study are included in the article/supplementary material, further inquiries can be directed to the corresponding author/s.

## Author Contributions

MRA, SN, MSM, ES, and VV: conceptualization, methodology, and investigation. MRA, SN, MAI, MM, and AH: software. MRA and SN: validation. MRA, SN, MSM, and AH: formal analysis. SN: resources and supervision. MRA, SN, MSM, ES, VV, and AH: data curation. MRA, SN, MSM, MAI, ES, VV, and MM: writing–original draft preparation. MRA, MH, ED, MA, MB, MS, SJ, MAI, and AH: writing–review and editing. SN, ES, and MH: project administration. SN, MH, ED, MA, and AH: funding acquisition. All authors have read and agreed to publish the current version of the manuscript.

## Conflict of Interest

The authors declare that the research was conducted in the absence of any commercial or financial relationships that could be construed as a potential conflict of interest.

## Publisher’s Note

All claims expressed in this article are solely those of the authors and do not necessarily represent those of their affiliated organizations, or those of the publisher, the editors and the reviewers. Any product that may be evaluated in this article, or claim that may be made by its manufacturer, is not guaranteed or endorsed by the publisher.

## Abbreviations

BARC: Bangladesh Agricultural Research Council
CTD: canopy temperature depression
EC: electrical conductivity
FC: field capacity
FKW: final kernel weight
KFD: kernel filling duration
KWLR: kernel weight ear^–1^ while increased kernel water loss rate
KDW: kernel dry weight
KFR: kernel filling rate
KGR: kernel growth rate
KU: Kasetsart University
KWC: kernel water content
MKWC: maximum kernel water content
PC: Pak Chong
PM: physiological maturity
SWD: stem weight depletion.
